# A 6-Year-Long Manipulation with Soil Warming and Canopy Nitrogen Additions does not Affect Xylem Phenology and Cell Production of Mature Black Spruce

**DOI:** 10.3389/fpls.2015.00877

**Published:** 2015-11-13

**Authors:** Madjelia C. E. Dao, Sergio Rossi, Denis Walsh, Hubert Morin, Daniel Houle

**Affiliations:** ^1^Département Productions Forestières, Institut de l’Environnement et de Recherches AgricolesOuagadougou, Burkina Faso; ^2^Département des Sciences Fondamentales, Université du Québec à ChicoutimiChicoutimi, QC, Canada; ^3^Direction de la Recherche Forestière, Forêt Québec, Ministère des Forêts de la Faune et des ParcsQuébec, QC, Canada; ^4^Ouranos, Consortium Sur la Climatologie Régionale et l’Adaptation aux Changements ClimatiquesMontréal, QC, Canada

**Keywords:** boreal forest, cambial activity, climate change, N deposition, intra-annual growth, increased soil temperature, wood anatomy, xylogenesis

## Abstract

The predicted climate warming and increased atmospheric inorganic nitrogen deposition are expected to have dramatic impacts on plant growth. However, the extent of these effects and their interactions remains unclear for boreal forest trees. The aim of this experiment was to investigate the effects of increased soil temperature and nitrogen (N) depositions on stem intra-annual growth of two mature stands of black spruce *[Picea mariana* (Mill.) BSP] in Québec, QC, Canada. During 2008–2013, the soil around mature trees was warmed up by 4°C with heating cables during the growing season and precipitations containing three times the current inorganic N concentration were added by frequent canopy applications. Xylem phenology and cell production were monitored weekly from April to October. The 6-year-long experiment performed in two sites at different altitude showed no substantial effect of warming and N-depositions on xylem phenological phases of cell enlargement, wall thickening and lignification. Cell production, in terms of number of tracheids along the radius, also did not differ significantly and followed the same patterns in control and treated trees. These findings allowed the hypothesis of a medium-term effect of soil warming and N depositions on the growth of mature black spruce to be rejected.

## Introduction

Surface temperature is projected to rise over the 21st century under all assessed emission scenarios (IPCC, 2014). Recent forecasts for the boreal forest of eastern Canada estimate increases of 3°C in mean annual temperature for the year 2050 (Plummer et al., 2006). Plant phenology, the timings of plant development and growth, is one of the traits sensitive to regional climate warming (Schwartz et al., 2006; Cleland et al., 2007). Apart from higher average temperatures, plants may also have to cope with other effects of global change, especially enhanced atmospheric nitrogen (N) deposition. Anthropogenic N depositions have greatly altered the N cycle and plant nutrition in the last two centuries and are projected to increase in the future (Thomas et al., 2010). In the boreal forest, plant growth is often considered to be limited by low temperatures and the availability of N (Reich et al., 2006). Thus, understanding the combined effects of warming and increased N depositions on xylem phenology, tree growth and the amount of cells produced, is critical for improving the prediction of tree responses to future climate.

The key role of soil and air temperatures in xylem phenology and cell production has recently been demonstrated (Rossi et al., 2008; Dufour and Morin, 2013). Soil temperature <6°C strongly inhibits xylem activity and water uptake in various conifers (Alvarez-Uria and Körner, 2007). Also, observations at the northern treeline showed no xylogenesis activity when soil temperature was < 3–5°C (Körner, 2003). Many studies underline the importance of soil temperature in defining the growing season (Körner, 2003; Alvarez-Uria and Körner, 2007). Kilpeläinen et al. (2007) reported that increased temperature resulted in thicker cell walls and higher wood density in Scots pine (*Pinus sylvestris* L.).

Studies on the effect of N deposition on plant growth revealed increased impact of N deposition on plant growth but decreased wood density and cell wall thickness in conifers (Hättenschwiler et al., 1996; Kostiainen et al., 2004).

The combined effect of warming and N fertilization can also be observed in xylem anatomy (Kostiainen et al., 2004; Kilpeläinen et al., 2007). Zhao and Liu (2009), by combining treatments of infrared warming and N deposition in China, obtained further increased performance of *P. tabulaeformis* seedlings but reduced that of *P. asperata*.

Most experimental investigations of N deposition and warming on plant growth have examined juvenile wood or used unrealistically high rates of N addition, so it is not possible to extrapolate the results to the conditions occurring in natural forest ecosystems. Few studies have used an accurate quantity of the additional N inputs that are expected in boreal forest ecosystems in the future, and together with soil warming or singly, the results indicated limited effects on the N status and growth rate after 3-year studies (Lupi et al., 2012; D’Orangeville et al., 2013).

As many factors affect tree growth patterns, short-term studies might be influenced by the confounding effect of several interacting environmental variables on plant growth (Battipaglia et al., 2015). Although these studies indicated no immediate effects after 3 years, we expected that cumulative effects of soil warming and increased N deposition would produce significant modifications in tree growth in the medium term. Few experiments have been done with concomitant variation of soil temperature and N deposition. Thus, the effects in the medium and long term on these environmental factors on trees, especially on cambium phenology and xylem cell production, remain largely unknown. To make realistic predictions of the comprehensive effects of climate change based on experiments a more complete understanding of the relationships between environmental factors (in terms of N deposition and increased temperature) and plant growth requires more years of manipulations.

In this study, we investigated if and how soil temperature and inorganic N deposition influence xylem phenology. We hypothesized that, in the medium term, increased soil temperature and N addition will enhance xylem production and cell differentiation and, in turn, increase the growth rate in boreal forests. To test this hypothesis, during 2008–2013, we used a unique experimental design in the field where inorganic N was repeatedly applied through artificial rain events on the tree canopy and the soil was warmed by 4°C with buried heating cables in two sites of the boreal forest of Québec, QC, Canada.

## Materials and Methods

### Study Site and Tree Selection

The study took place in two mature even-aged stands of black spruce *[Picea mariana* (Mill.) BSP] located at different latitudes and altitudes in the boreal forest of Quebec, Canada. The more northern site Bernatchez (abbreviated as BER) is located near Lac Bernatchez, in the Monts-Valin (48°51′N, 70°20′W, 611 m a.s.l.) while the other Simoncouche (SIM) is in the Laurentides Wildlife Reserve, within the Simoncouche research station (48°13′N, 71°15′W, 350 m a.s.l.). Both regions are included in the balsam fir-white birch ecological domain (Saucier et al., 1998), with an understorey vegetation mainly composed of *Kalmia angustifolia* L., *Ledum groenlandicum* Oeder, *Cornus canadensis* L., *Vaccinium myrtilloides* Michx., and soil vegetation of *Sphagnum* sp. and mosses [*Hylocomium splendens* (Hedw.), *Pleurozium schreberi* (Brid.), *Ptilium crista-castrensis* (Hedw.) De Not.]. The soil in both regions is podzol with a mor-type humus (Rossi et al., 2015). The mean annual temperature is 0.3 and 2.0°C at BER and SIM. From May–September mean annual rainfall is 401.8 and 425.4 mm, at SIM and BER, respectively. SIM derived from a forest fire in 1922, while the forest fire at the origin of the stand in BER has been estimated to have occurred between 1865 and 1870. The stands are growing on gentle slopes (8–17%) and drained glacial tills.

In each site, six co-dominant trees were chosen with upright stem, healthy overall appearance and similar growth patterns. The homogeneity in growth rates was assessed during a preliminary investigation by extracting wood cores and counting the number of tracheids along three previous tree rings (Rossi et al., 2007). The average diameter at breast height and the average height of sampled trees were 17 ± 2 and 21 ± 4 cm, and 15 ± 2 and 14 ± 2 m, at BER and SIM, respectively.

### Experimental Design

In each site, two treatments were combined: an increase in soil temperature (H-treatment) and a canopy application of artificial rain enriched with nitrogen (N-treatment). The combination of the treatments resulted in four experimental groups: heated only trees (H), N-enriched only trees (N), heated, and N-enriched trees (NH) and control trees, for which the soil was not heated and that received no N-enrichment (C). The two treatments were attributed randomly to experimental trees resulting in a random split plot design with three replications.

For the H-treatment, heating cables were installed during autumn 2007 between the organic and mineral soil layers, at about 20 cm depth, where the majority of the root system of black spruce is localized (Ruess et al., 2003), following a spiral pattern at a distance of 90–200 cm from the stem collar. Cables were laid by cutting the soil vertically with a shovel or a knife and manually inserting the cable in the resulting narrow “trench”, which was then rapidly reclosed. To account for potential root damage and soil disturbance during cable laying, non-heating cables were also installed around non-heated trees (C and N).

Power was supplied by a diesel generator located at 200 m from the site. H treatment consisted of increasing the soil temperature by 4°C during the first part of the growing season. This led to an earlier snowmelt and an increase in annual soil temperature in agreement with the estimates for 2050 by the FORSTEM climatic model developed for the boreal forest of eastern Canada (Houle et al., 2012). Heating started on different dates according to year and site, usually with a 2 weeks delay between SIM and BER to reflect the difference in temperature between the two sites (Lupi et al., 2010) and achieve a 1–2 weeks earlier snowmelt in heated plots. Soil temperature was measured between the coils of the cables around three heated and three control trees.

The temperature differential between control and treated trees was maintained during April–July, the period in which most cambial division takes place (Thibeault-Martel et al., 2008), to reproduce an earlier snow melt and a longer snow free period. Soil temperature was measured every 15 min and data were stored as hourly averages in CR1000 dataloggers (Campbell Scientific Corporation, Canada). Volumetric water content of heated and non-heated plots was measured in July 2009 using a CS-616 probe (Campbell Scientific Corporation, Canada) mounted on a portable device to check for differences in soil moisture content. No significant difference was found between heated (H and NH) and non-heated trees (C and N) (Lupi et al., 2012).

Artificial rain was produced by sprinklers installed above the canopy of each tree. Each week, the equivalent of 2 mm rainfall was applied to the canopy, during the frost-safe period (June to September), for a number of weeks varying between 12 and 14. Rain was applied over a circular 3-m radius area centered on the stem of each experimental tree, which allowed the canopy area to be covered. Non-N-enriched trees (C and H) were irrigated with a water solution reproducing the chemical composition of natural rainfall at the studied sites (Duchesne and Houle, 2006, 2008), while for N-enriched trees (N and NH), a threefold increase in ammonium nitrate (NH4NO3) concentration was used (**Table 1**).

**Table 1:** Ion concentration in the artificial rain.

Ion	Control (μequiv. L^-1^)	N-treatment (μequiv. L^-1^)
Na^+^	2.24	2.24
Ca^+2^	5.00	5.00
Mg^+2^	1.66	1.66
K^+^	0.76	0.76
H^+^	16.18	16.18
Cl^-^	2.24	2.24
SO4^-2^	23.69	23.69
NH^4+^	14.93	44.78
NO^3-^	14.93	44.78

It is expected that frequent artificial rain additions directly to the canopy, with relatively low inorganic N concentration, better imitate the way anthropogenic derived N depositions are reaching boreal forest ecosystems than massive soil applications do.

### Meteorological Data

At each site, a standard weather station was installed in a forest gap to measure air, soil temperature and snow depth. The soil temperature was measured both on the mineral and organic layers, at 20–30 and 5–10 cm depth, respectively, and air temperature at a height of 2 m. Snow depth was measured with an acoustic distance sensor that quantifies the elapsed time between emission and return of an ultrasonic pulse and automatically corrects for variations of the speed of sound during the year using the measurements of air temperature. Data were collected every 15 min and stored as hourly averages in CR10X dataloggers (Campbell Scientific Corporation, Logan, UT, USA). Daily mean values were later calculated for further analysis.

### Sample Collection and Preparation

During 2008–2013, microcores (2.5 mm diameter and 25 mm long) were collected weekly from the stem from April to October with a Trephor (Rossi et al., 2006a) following a counterclockwise-elevating spiral centered at breast height. In SIM and BER, samples were collected weekly in early summer (May–June) and every 2 weeks during July–October. Micro-cores usually contained the previous 5–10 tree rings and the developing annual layer with the cambial zone and adjacent phloem tissues. Wood samples were always taken at 5–10 cm intervals to avoid the formation of resin ducts as a reaction to disturbance. The micro-cores were placed in Eppendorf micro-tubes containing a water:ethanol solution (1:1). Microcores were dehydrated through successive immersions in ethanol and Histosol and embedded in paraffin (Rossi et al., 2006b). Transverse sections 6–10 mm thick were cut with a rotary microtome, stained with cresyl violet acetate (0.16% in water) and observed within 20–30 min under visible and polarized light at magnifications of 400–500× to differentiate the cambium and developing xylem cells. The cambial zone and cells in radial enlargement showed only a primary wall, which, unlike the secondary wall, did not shine under polarized light (Gričar et al., 2006). Cambial cells were characterized by thin cell walls and small radial diameters, while enlarging cells had a radial diameter at least twice that of a cambial cell. Cells in wall thickening shone under polarized light and during the maturation process showed a coloration varying from light to deep violet. As lignification advanced, a blue coloration starting from the cell corners spread into the secondary walls. Since lignin deposition may persist after the end of cell wall thickening (Gindl et al., 2000), cells were considered mature when the violet was completely replaced by blue (Rossi et al., 2006b; Rossi et al., 2014).

The number of cells in each phase was counted along three radial rows and the total number of xylem cells was calculated as the sum of cells in radial enlargement and wall thickening and lignification and mature cells. In spring, xylem formation was considered to have begun when the average number of cells in enlarging phase between the three radial rows was more than one. In late summer, when no further cells were observed undergoing wall thickening and lignification, xylem formation was considered complete.

The phenology of xylem development was assessed for each tree. Four phenophases, computed in days of the year (DOY), were considered, including onset and ending of both cell enlargement and wall thickening and lignification. Duration of xylem formation was calculated as the difference between the onset of cell enlargement and the ending of lignification.

### Statistical Analysis

Analysis of variance for repeated measures (ANOVAR) in a split-plot mixed design was used for all variables of phenology and cell production, with site as block factor, treatments N × H as main plots, and Year as repeated factor. As sampling times were correlated, the selection of the covariance structure was based on the lower Akaike’s information criterion (AIC). The first-order autoregressive [AR(1)] provided the suitable correlation structure (Wolfinger, 1993). ANOVAR was performed with the MIXED procedure of JMP Pro 11.1.1 (SAS Institute, Cary, NC, USA). Normality and homoscedasticity were graphically verified on residual plots of the linear models (Quinn and Keough, 2002).

## Results

### Temperature and Snow Depth

**FIGURE 1:**
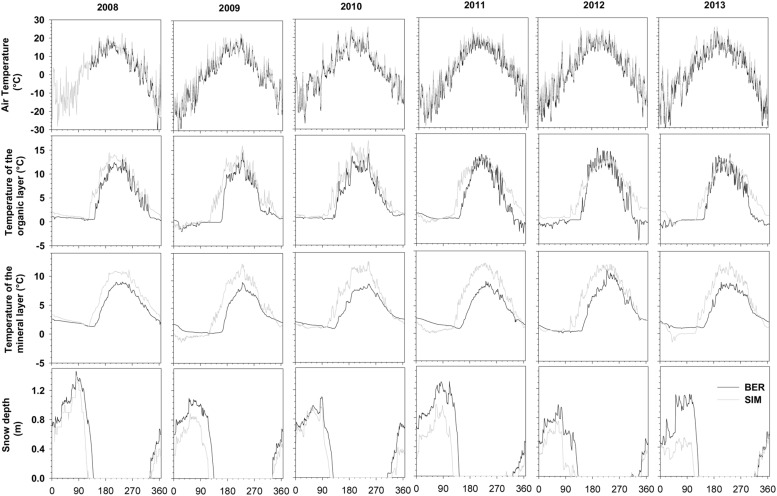
**Daily mean temperature and snow depth recorded at BER and SIM during 2008–2013**.

The sites were characterized by long winters with temperatures close to or below zero from October to April. BER, located at the higher latitude and altitude, was the coldest site in winter with the absolute minimum temperatures being measured in 2013 reaching -41.63°C (**Figure 1**). The average temperature for the period ranging between DOY 122–273 (May–September) in BER was two degrees lower than in SIM (11.8°C vs. 13.8°C). Summers were short, with absolute maximum temperatures reaching 26°C in 2010 (**Figure 1**).

During 2008–2013, mean temperatures in the organic and mineral layers varied between 3.4°C and 5.5°C. During winter, soil temperature remained below 3°C and was always lower at BER (**Figure 1**). Summer temperatures in the organic and mineral soil reached 10–15°C, starting to increase only after snowmelt (**Figure 1**). Maximum absolute snow depth varied between 1.0 and 1.5 m, with 2012 being the year with the least snow depth (**Figure 1**). The moment of snowmelt varied between years, but, on average, occurred 15 days later in BER.

### Xylogenesis

**FIGURE 2:**
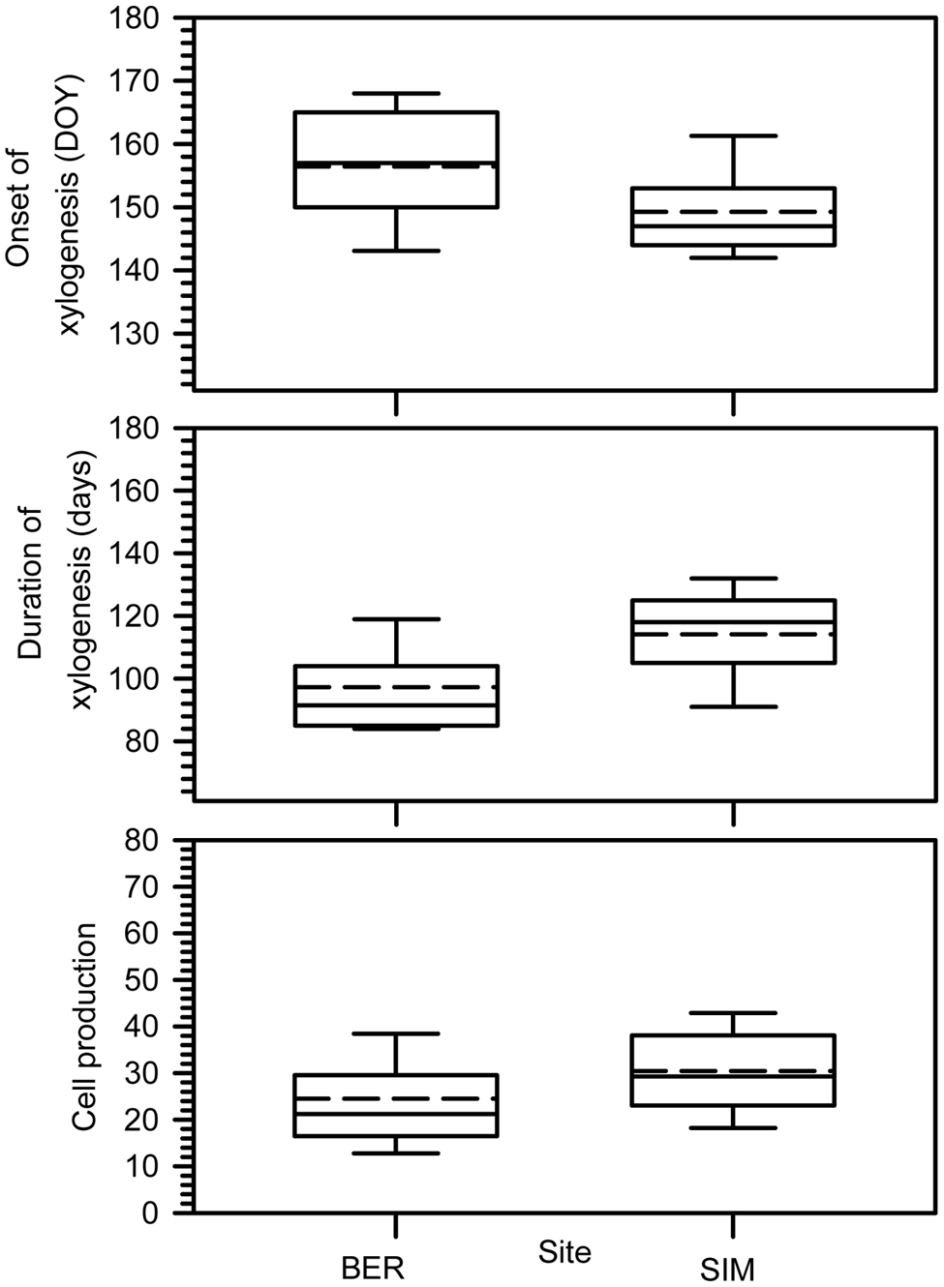
**Onset, duration of xylogenesis and cell production.** Boxes represent upper and lower quartiles, whiskers achieve the 10th and 90th percentiles and the median and mean are drawn as horizontal solid and dashed lines.

The onset of xylogenesis started from late May to mid-June (DOY 146–166) (**Figure 2**). The onset of xylem growth was observed later in BER, the colder site. Xylogenesis lasted 95–120 days on average; SIM clearly had the longest duration of xylogenesis, reaching 121 days. Cell production, corresponding to the number of cells produced along the tree ring, differed between the two sites (**Figure 2**). On average, SIM had the highest cell productions and the longest period of growth, with 39 cells.

### Differences between Sites

**FIGURE 3:**
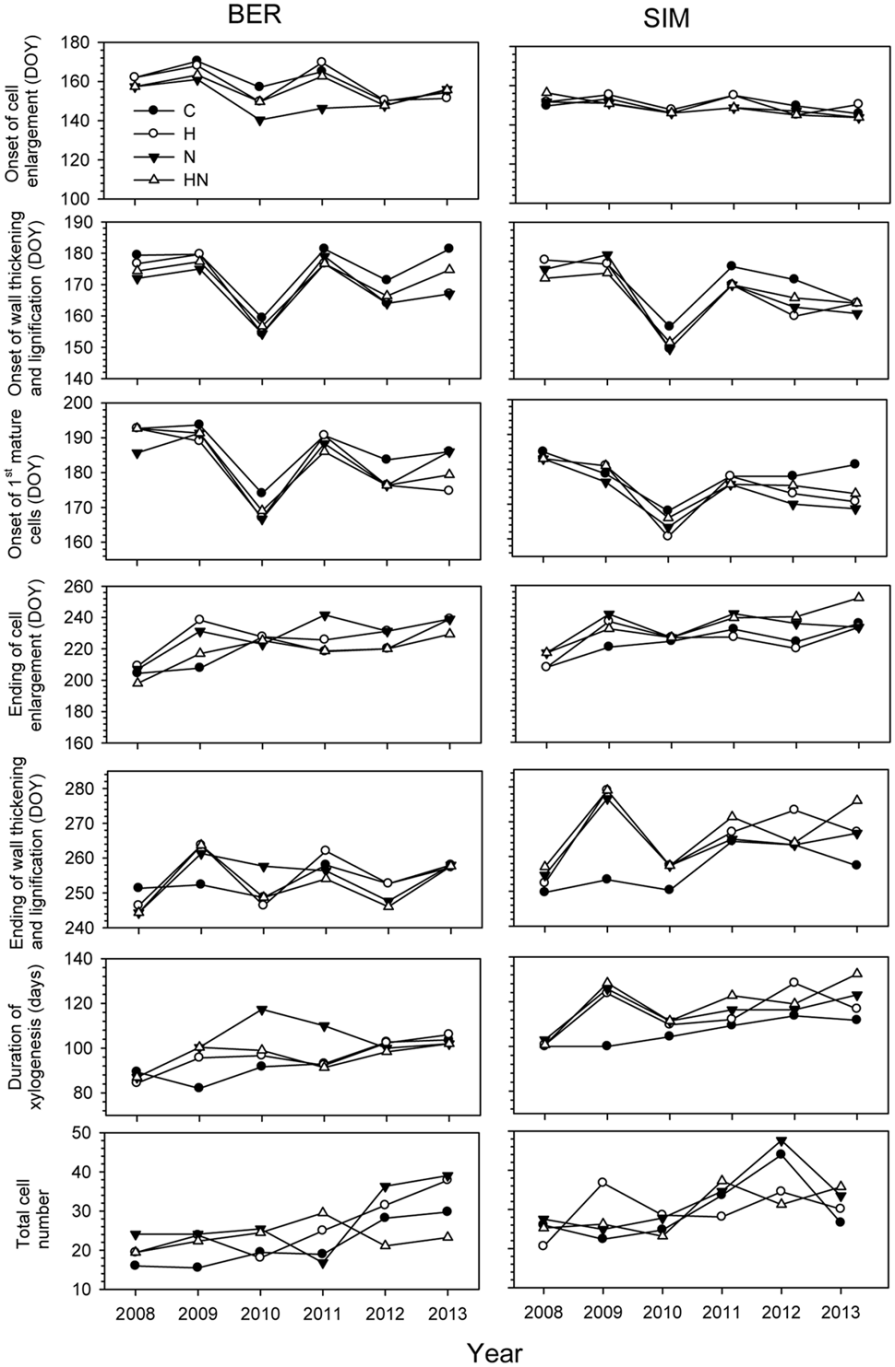
**Phenological phases of xylem differentiation, duration, and total mature cells of xylogenesis in C (control), H (heated), N (nitrogen), and HN (heated and nitrogen) trees in BER and SIM during 2008–2013**.

The ANOVAR test detected a significant difference in onset of cell enlargement (*P* < 0.05) between sites (**Table 2**). The onset occurred between DOY 140 and 170 and between DOY 146 and 160 at BER and SIM, respectively (**Figure 3**). It began 1 week earlier in BER, but the latest onset of cell enlargement and the greatest inter-tree variability during the 6 study years were also observed in BER (**Figure 3**). The dynamics of the onset of cell wall thickening was similar in both sites for all treatments but the model showed a significant effect of site (*P* < 0.001), with trees starting wall thickening 11 days earlier in SIM than in BER (DOY 148 and 159, respectively) (**Figure 3**). The onset of cell maturation was significantly different between sites (*P* < 0.01) (**Table 2**) with SIM being the earliest (**Figure 3**). Even if, on average, cell enlargement ended 10 days earlier at BER (DOY 198) than at SIM (DOY 208), no statistically significant difference was found between sites (*P* > 0.05) (**Table 2**). The duration of xylogenesis was significant between sites (*P* < 0.01) (**Table 2**), with the whole process being completed in 35 days (between DOY 82 and 117) in BER and 32 days (between DOY 100 and 132) in SIM (**Figure 3**). There was no significant difference for cell production between sites (*P* > 0.05) (**Table 2**). On average, trees at the SIM site produced more cells in the tree ring, with the highest and lowest values of 48 and 20 cells observed in 2008 and 2012, respectively (**Figure 3**).

**Table 2:** *F*-values of the mixed procedure with repeated measurements using Site as block factor, Year as repeated factor for the different phases of xylem phenology and cell production.

Source of variation	Onset of cell enlargement	Onset of wall thickening and lignification	Onset of cell mature	Ending of cell enlargement	Ending of wall thickening and lignification	Duration of xylogenesis	Total cell number
Site	7.83^∗^	27.08^∗∗∗^	26.11^∗∗∗^	2.27	12.06^∗∗^	11.73^∗∗^	2.29
*N*	0.21	0.84	0.89	0.01	1.24	0.16	0.02
Site × N	0.04	0.00	0.41	0.11	1.50	0.63	0.04
T	2.18	1.59	1.49	1.39	0.43	1.30	0.20
Site × T	0.40	0.09	0.00	1.76	0.90	0.04	0.00
N × T	0.27	2.53	3.44	1.78	0.67	0.54	0.59
Site × N × T	0.40	0.47	0.06	2.04	0.07	0.03	0.14
Year	10.93^∗∗∗^	57.88^∗∗∗^	51.51^∗∗∗^	17.37^∗∗∗^	14.60^∗∗∗^	7.46^∗∗∗^	11.36^∗∗∗^
Site × Year	2.07	2.74^∗^	2.97^∗^	0.64	1.42	1.88	2.73^∗^
N × Year	0.60	0.24	1.49	0.66	1.60	1.37	4.33^∗∗^
Site × N × Year	0.73	0.41	0.92	0.40	0.14	0.19	1.63
T × Year	1.26	0.20	0.24	0.73	2.16	1.87	0.47
Site × T × Year	0.82	0.16	0.83	0.33	0.52	0.87	1.38
N × T × Year	0.26	1.47	1.13	2.25	1.03	0.70	2.32
Site × N × T × Year	0.44	0.93	0.36	0.61	0.39	0.63	1.05

*The treatments are reported as N (nitrogen), and H (soil warming), and N × H (interaction between N and H).*

*^∗^p < 0.05; ^∗∗^p < 0.01, and ^∗∗∗^p < 0.001.*

### Differences between Years

Years had highly significant effects on xylem phenology and cell production (*P* < 0.001) and significant effects of the interactions between site and years were also observed for the onset of wall thickening, the mature cell and cell production (*P* < 0.05) (**Table 2**). The dynamics of xylem formation remained similar in each xylem phenological phase. No significant effect on phenological phase was found with the N treatment but the interaction of N × year was significant (*P* < 0.01). An exception in the year 2010 was observed in the onset of cell wall thickening, which occurred markedly earlier, in late May (DOY 148) (**Figure 3**). The onset of cells maturation also appeared earlier in 2010 (**Figure 3**).

The ending of cell enlargement was observed between mid-July and late August (DOY 198–242) in all sites excepted in 2013 when it occurred later at SIM (mid-September, DOY 252) (**Figure 3**). The ending of cell thickening occurred in late September for all treatments in both sites during 2008–2013, except in 2009 in SIM, where the highest variations between treatments were observed (40 days between control and the other treatments) (**Figure 3**).

During 2008–2013, important variations occurred in the number of cells produced, with the highest variability and the greatest number of cells produced in BER and SIM in 2012 and 2013 (**Figure 3**). In 2013, xylogenesis started 18 days earlier in BER on DOY 82, and ended 15 days earlier on DOY 117, thus the duration of growth was markedly longer in BER (**Figure 3**).

### Effects of the Treatments

No significant effect of the treatments was found (**Table 2**). The onset of cell wall thickening occurred between mid- (DOY 170) to late- (DOY 181) June for all treatments, with no significant treatment effects (**Table 2**). The treatments also had no significant effect on the moment at which the first mature cells were formed (**Table 2**). The ending of cell enlargement occurred at the same time for all treatments and ANOVAR showed no significant difference between treatments (**Table 2**). It was observed between mid-July and late August (DOY 198–242) in both sites (**Figure 3**). The last cells in wall lignification, which corresponded to the ending of xylem differentiation, occurred between late August and mid-October, with SIM being the later site to complete differentiation (**Figure 3**). ANOVAR performed on the ending of cell wall thickening and lignification showed no significant effect of the treatments (**Table 2**). It was not possible to find significant effects of the treatments on the overall period for completing process of xylogenesis (**Table 2**).

## Discussion

This study conducted in two matured black spruce stands of the boreal forest of Quebec, Canada tested the hypothesis that xylem phenology and cell production were affected by increased soil temperature and inorganic N availability in precipitation. Soil temperature was increased by 4°C during the first part of the growing season and precipitations containing three times the current inorganic N concentration in ambient precipitation were repeatedly applied during the growing season from 2008 to 2013. The experiment consisted of frequent canopy applications of inorganic N at realistic concentrations with the aim of simulating future rain composition. After a 6-year experiment, our results showed no substantial change between treatments in xylem phenology and cell production, so our hypothesis had to be rejected. However, a potential effect of soil warming and increased N deposition could still occur under a longer period of experimentation.

In previous studies, localized warming of the stem often increased cell productions but only in the zone of treatment application (Gričar et al., 2006; Gričar et al., 2007). Soil warming significantly enhanced diameter growth of woody individuals, especially shrubs (Farnsworth et al., 1995). McWhirter (2013) investigated the responses of *Malus coronaria* (Crap apple) seed germination and seedling growth to warming and nitrogen in old temperate forests and in greenhouse and the results suggested direct effects on germination and establishment of seedlings. In northern China, the warming response of plant phenology (including flowering and fruiting date as well as reproductive duration) is larger in earlier than later flowering species in temperate grassland systems, but no interactive effect between warming and N addition was found on any phenological event (Xia and Wan, 2013).

In contrast to our results, Lupi et al. (2012) by combining early season soil warming with canopy applications of water containing N at a concentration three times higher than the ambient precipitation in the same sites, detected in the short term, that soil warming resulted in earlier onset and extended duration of xylogenesis in the root and along the stem. The results of Lupi et al. (2012) were not confirmed on our longer period of observations. Melillo et al. (2011) reported carbon gains in the woody tissues of trees in a 7-year soil warming study in a mixed hardwood forest ecosystem. (Overdieck et al., 2007) also recorded increased stem diameter, stem height and stem mass for beech seedlings grown for 2.5 years at increased air temperature.

The absence of growth stimulation in our study, which used realistic increased concentrations of inorganic N concentration in precipitation, suggests that growth stimulation (due to increasing N availability) is not to be expected in the future for the boreal forest of eastern Canada. However, given the experimental conditions used and the gaps in our understanding of N foliar uptake, it would be premature to definitely conclude an absence of effects. For instance, in a labeling experiment with ^15^N, less than 5% of the label was recovered in live foliage and wood after 2 years of N addition to the canopy with a helicopter (Dail et al., 2009). The majority of the label was recovered in twigs and branch materials. Thus, most of the N was retained on plant surfaces, branches and main-stem bark, with little being assimilated into foliage that could then be transported to new forming cells in the stem. In good agreement with the latter study, another recent study involving canopy application of inorganic ^15^N in a coniferous stand (Gélinas-Pouliot, 2013) has shown that small twigs, not needles, was the main sink for the added N.

It is, however, possible that the N scavenged by the twigs may take a certain time to reach the stem where cell divisions occur and that a N effect could be observed with a longer period of experimentation. It has also been suggested that changes in nutrient cycling due to increased N deposition and its potential effect on tree growth, may become significant only in the medium and long term, since trees seem less receptive than other plants and microorganisms to the uptake of inorganic and organic N in the short term (Näsholm et al., 2009). Thus, although a direct effect of earlier snowmelt and higher soil temperature on tree growth does not appear likely based on our results, an effect of N addition could potentially appear with longer term addition.

Our results revealed the impact of time scale on xylem phenology and cell production. As shown in **Figure 3** and **Table 1**, the onset of cell wall thickening and the first mature cell had already started earlier in 2010 at the end of May when snowmelt had just finished and air temperature reached values above zero. This led to the conclusion that the onset of wood formation can be affected by snowmelt and temperatures (Rossi et al., 2011a; Dufour and Morin, 2013). Based on studies of conifers across a wide range of different geographical locations, Rossi et al. (2008) found that air temperature is also a critical factor limiting the differentiation of xylem cells.

During the 6 years of treatments, the maximum amount of 48 cells produced occurred in 2012, the least snow depth and warmest year. Several authors had found that the onset and ending increments are affected by an air temperature threshold (Deslauriers et al., 2008; Dufour and Morin, 2010) as well as snowmelt and soil temperature (Rossi et al., 2011b; Lupi et al., 2012). Kalliokoski et al. (2012) indicated that weather variation induced differences of up to 28 days in the onset of tracheid formation between years for Norway spruce. Mäkinen et al. (2003) also revealed that interannual variations in increment onset can be important. The investigations on montane Mediterranean tree species (*Cedrus libani)* at different altitudes reported differences in onset, duration and end of cambial activity and xylogenesis as well as growth rates with respect to temperature, especially daily means of air and stem temperature (Guney et al., 2015).

In the medium term, despite some observed significant effects, the results showed similar dynamics between sites for xylem phenology for all treatments. The differences often observed between sites may therefore rather indicate that xylem phenology and cell production are controlled by temperature and snowmelt. Thus the differences in timing can be explained by the lower average air temperature at the BER site.

## Conclusion

The two boreal forest sites studied showed significant differences for xylem phenology of black spruce, except for the ending of cell enlargement and cell production, which could be attributed to their difference in average annual air temperature.

It was, however, found that a 6-year experiment of soil warming and increased inorganic N additions applied directly to the canopy failed to induce significant differences in xylem phenology and cell production at both sites, which allowed our hypothesis to be rejected. Different results could be expected with a longer-term experiment. For instance, it is possible that the N added to the canopy could be slowly translocated from the twigs to the dividing cells and that could lead to an increase in growth in the longer term.

## Conflict of Interest Statement

The authors declare that the research was conducted in the absence of any commercial or financial relationships that could be construed as a potential conflict of interest.
